# Dataset of the proteome of purified outer membrane vesicles from the human pathogen *Aggregatibacter actinomycetemcomintans*

**DOI:** 10.1016/j.dib.2016.12.015

**Published:** 2016-12-15

**Authors:** Thomas Kieselbach, Jan Oscarsson

**Affiliations:** a Department of Chemistry, Umeå University, Umeå, Sweden; b Oral Microbiology, Department of Odontology, Umeå University, Umeå, Sweden

**Keywords:** Micobiology, Odontology, Periodontitis, Outer membrane vesicle, Proteomics

## Abstract

The Gram-negative bacterium *Aggregatibacter actinomycetemcomitans* is an oral and systemic pathogen, which is linked to aggressive forms of periodontitis and can be associated with endocarditis. The outer membrane vesicles (OMVs) of this species contain effector proteins such as cytolethal distending toxin (CDT) and leukotoxin (LtxA), which they can deliver into human host cells. The OMVs can also activate innate immunity through NOD1- and NOD2-active pathogen-associated molecular patterns. This dataset provides a proteome of highly purified OMVs from *A. actinomycetemcomitans* serotype *e* strain 173. The experimental data do not only include the raw data of the LC-MS/MS analysis of four independent preparations of purified OMVs but also the mass lists of the processed data and the Mascot.dat files from the database searches. In total 501 proteins are identified, of which 151 are detected in at least three of four independent preparations. In addition, this dataset contains the COG definitions and the predicted subcellular locations (PSORTb 3.0) for the entire genome of *A. actinomycetemcomitans* serotype *e* strain SC1083, which is used for the evaluation of the LC-MS/MS data. These data are deposited in ProteomeXchange in the public dataset PXD002509. In addition, a scientific interpretation of this dataset by Kieselbach et al. (2015) [2] is available at http://dx.doi.org/10.1371/journal.pone.0138591.

**Specifications Table**

**Table:** 

Subject area	*Biology.*
More specific subject area	*Micobiology, odontology, infection biology.*
Type of data	*Mass spectrometry raw files (Waters), text files, Mascot.dat files, Excel tables*.
How data was acquired	*Mass spectrometry (LC-MS/MS, DDA) using a Synapt G2 instrument from Waters linked on-line to a nano UPLC (Waters). In silico* analysis of the dataset.
Data format	*Raw and processed.*
Experimental factors	*Not applied.*
Experimental features	*Qualitative protein analysis of purified bacterial outer membrane vesicles*
Data source location	*Umeå University, Umeå, Sweden.*
Data accessibility	*Data is at ProteomeXchange*[1]: PXD002509.

**Value of the data**

• This dataset may be useful for the design of targeted mass spectrometry assays to quantify outer membrane vesicle proteins in *A. actinomycetemcomitans* strains and in other bacterial species.
• This dataset lays molecular groundwork for the design of experiments to disclose virulence-related functions of *A. actinomycetemcomitans* OMVs, and mechanisms of how proteins with preferential cytoplasmic localization might be targeted for vesicle export in bacteria.
• Finally, this dataset may be of value to address post-translational modifications of outer vesicle proteins.

## 1 Data

The core of this dataset is the raw and processed data of the LC-MS/MS analysis (DDA) of four independent preparations of purified OMVs of *A. actinomycetemcomitans* strain 173 and their evaluation. The processed dataset contains 48,538 mass spectra (Mascot Distiller 2.5; Matrixsciene) that allow identification of 501 non-redundant proteins out of which 151 proteins are detected in at least three of four preparations. The number of proteins identified in the individual OMV preparations is as follows: 75 proteins in preparation 1, 206 proteins in preparation 2, 488 proteins in preparation 3 and 228 proteins in preparation 4. In addition, the dataset includes a bioinformatics analysis of the complete genome of *A. actinomycetemcomitans* serotype e strain SC1083, which provides all COG annotations and predicted subcellular locations (PSORTb 3.0) as a tool for further evaluation of the data. Kieselbach et al. [2] performed a scientific interpretation of this dataset with the goal to identify potential new OMV proteins that may contribute to human disease.

## 2 Experimental design, materials and methods

**Fig. 1:**
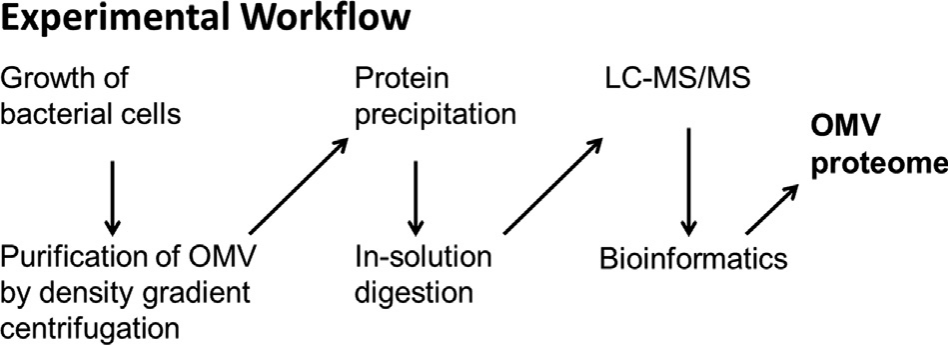
Scheme of the experimental workflow used for the purification and analysis of the outer membrane vesicles of *A. actinomycetemcomitans* serotype e strain 173. In total, four preparations were analyzed to ensure good coverage of the outer membrane vesicle proteome and reproducibility of the protein identifications.

The work has the goal to provide a dataset that is suitable to define the proteome of OMVs of the human pathogen *A. actinomycetemcomitans* strain 173 and that to identify proteins that are suitable candidates for further studies of host cell infection. Having access to OMVs of high purity is essential to achieve this goal. To obtain an OMV fraction of high purity, the experimental design includes a tandem purification, in which OMVs first are enriched by differential centrifugation and then further purified by density gradient centrifugation. The subsequent workflow adds a proteomics pipeline, in which the OMV proteins are digested using trypsin and then identified through LC-MS/MS (DDA) and bioinformatics. The different steps of this workflow are outlined in Fig. 1. To ensure reproducibility of the protein identifications and good coverage of the OMV proteome, this workflow was applied to four independent preparations of purified OMVs. The benefit of this design is a highly purified OMV fraction that provides sufficient material for a protein analysis by mass spectrometry. However, the high purity of the OMV fraction comes at the cost of low yields, and the tandem purification of this work does not provide enough material for a quantitative comparison of OMVs under different physiological conditions.

### 2.1 Bacterial strains and growth conditions used

The *A. actinomycetemcomitans* serotype e strain 173 is a rough colony type strain and belongs to a collection of strains that was sampled from an adolescent population in West Africa. It was isolated from a person who had periodontal attachment loss at the baseline [3]. The *A. actinomycetemcomitans* strain was routinely cultivated in air supplemented with 5% CO_2_ at 37 °C on blood agar plates (5% defibrinated horse blood, 5 mg hemin/l, 10 mg Vitamin K/l, Columbia agar base) as described [4].

### 2.2 Isolation and purification of outer membrane vesicles (OMVs)

Crude OMVs were prepared by differential centrifugation using *A. actinomycetemcomitans* cells, which were harvested from ten blood agar plates [5], [6]. Briefly, the OMVs were separated from the harvested bacteria by centrifugation at 8000×*g* and 4 °C for 30 min (JA-25.50 rotor Beckman Coulter, Bromma, Sweden). The supernatants were filtered through 0.22 μm membranes to remove cell fragments (Merck Millipore, Solna, Sweden), and the fraction of crude OMVs was collected by ultra-centrifugation for 2 h at 85,000×*g* and 4 °C (70 Ti rotor, Beckman Coulter). Subsequently, the OMV pellets were washed two times with PBS (85,000×*g*; 2 h, 4 °C) and suspended in PBS. The yield of OMVs was estimated by measuring their protein content at 280 nm using a Picodrop™ (Picodrop Ltd.) [6]. To assess the uniformity of the OMV preparations, the fraction of crude OMVs was analyzed by atomic force microscopy (AFM) and SDS-PAGE. In addition, the crude OMVs were tested for lack of bacterial contamination by cultivating small aliquots on blood agar in air supplemented with 5% CO_2_, at 37 °C for 3 days. To separate the OMV preparations from free or loosely associated proteins, they were further purified by density gradient centrifugation in Optiprep medium (Sigma Aldrich, Stockholm, Sweden) [5], [6]. In this step, the OMVs migrate to positions equal to their density, and only outer membrane proteins, and proteins that are enclosed in the OMVs co-migrate with the OMVs in the density gradient [7]. The OMV pellets were resuspended in 50 mM HEPES (pH 6.8) and mixed with OptiPrep (Sigma-Aldrich) to a final concentration of 45% (v/v) OptiPrep. The final volume was 150 μl. The sample was transferred to the bottom of a 4-ml ultracentrifuge tube and overlayed stepwise with layers of Optiprep in 50 mM Hepes (pH6.8) of decreasing density: 900 μl of 35%, 900 μl of 30%, 660 μl of 25%, 660 μl of 20%, 400 μl of 15%, and 500 μl of 10%. The gradients were centrifuged at 180,000×*g* (4 °C, 3 h) in an SW 60 Ti rotor (Beckman Coulter), and fractions of equal volumes (200 μl) were removed sequentially from the top. The individual fractions were assessed for their protein composition using SDS-PAGE, and the fractions containing the purified OMVs were stored in −20 °C prior to further analysis.

### 2.3 Preparation of in-solution digests of OMV proteins

The fractions with density-gradient purified OMVs were pooled, which resulted in a final volume of 780 µl. Subsequently, 400 µl HEPES buffer (50 mM, pH 7.8) was added to increase the pH to a value above 7. For reduction of the disulfide bonds, dithiotreitol was added at a final concentration of 50 mM, and the sample was heated for 20 min at 60 °C. For the alkylation of thiol groups, a fresh solution of 0.55 M iodoacetamide (IAM) was added to a final concentration of 20 mM and allowed to react for 60 min in the dark. To remove remaining amounts of Optiprep and other reagents, the OMV proteins were precipitated overnight in −20 °C using trichloroacetic acid. The next day, the OMVs were collected by centrifugation at 16,000×*g* for 30 min at 4 °C using a JA 18.1 rotor and a Beckman Coulter Avanti J-20 XP centrifuge (Beckman Instruments Inc., California, USA). The OMV pellet was washed using 80% ethanol, and the OMVs were collected again by centrifugation at 16,000×*g* for 15 min at 4 °C. Ultimately, the OMV pellet was air-dried used for the preparation of an in-solution digest to create peptides for mass spectrometry analysis.

For solubilization, the OMV pellet was suspended in 15 μl fresh 8 M urea and 20 μl of 50 mM ammonium bicarbonate containing 0.2% ProteaseMax^TM^ surfactant (Promega Biotech, Nacka, Sweden), and the suspension was shaken at 150 rpm for 20 min at 37 °C. Subsequently, the following solutions were added: 50 μl of 50 mM ammonium bicarbonate, 10.4 μl of Milli Q water, 1 μl of 50 mM ammonium bicarbonate containing 1% ProteaseMax surfactant (Promega Biotech, Nacka, Sweden), and 3.6 μl of 0.5 μg/μl trypsin stock solution (sequencing grade trypsin, Promega Biotech, Nacka, Sweden). The final concentrations were 40 mM ammonium bicarbonate, 0.05% ProteaseMax surfactant, 1.2 M urea and 18 ng/ml of trypsin in a volume of 0.1 ml. The digestion with trypsin was carried out for either 1–1.5 h at 50 °C or for 2–3 h at 37 °C [8]. The digestion was terminated upon addition of 10% trifluoroacetic acid to a final concentration of 0.5–1.0%, and the peptides were desalted using homemade reversed phase micro columns packed with C_18_ filters and Poros R3 material [9], [10]. The bound compounds were eluted using 0.1% formic acid containing 50% acetonitrile. The solvent was removed using a speedvac, and the dried in-solution digest sample was dissolved in 0.1% formic acid for further analysis by mass spectrometry.

### 2.4 LC-MS/MS analysis and data processing

The analysis of the in-solution digest samples was achieved using LC-MS/MS (DDA, 5 MS/MS channels) with a Synapt G2 mass spectrometer (Waters, Sollentuna, Sweden) that was linked to a nano UPLC (Waters, Sollentuna, Sweden). Separation of the peptides was performed by C_18_ nano reversed phase chromatography (Acquity nano UPLC column 1.8 mm HSS T3 75 mm×200 mm). The peptides were separated at a flow rate of 300 nl/min using a 4 h long linear gradient (1–30 percent acetonitrile for 3 h, 30–50 percent acetonitrile for 1 h). Spectra were processed using the ProteinLynx Global Server 2.5.2 software (Waters, Sollentuna, Sweden) with lockspray calibration and fast de-isotoping for the MS and MS/MS mode. In addition, the spectra were also processed using the Mascot Distiller (version 2.4.3.3, Matrix Science, London, UK) and the standard settings for DDA data from Waters instruments. Database searches using the peaklist files of the processed mass spectra were performed using the Mascot search engine (version 2.4, MatrixScience, London, UK) in the database of *A. actinomycetemcomitans*serotype e strain SC1083, which is available at Ensembl Bacteria at the URL: http://bacteria.ensembl.org/aggregatibacter_actinomycetemcomitans_serotype_e_str_sc1083/Info/Index. The reason for using the database of another serotype e strain was that the genome of strain 173 is not available. The parameters for the database searches permitted mass errors of 20 ppm (MS mode) and 0.1 Da (MS/MS mode), respectively. Modifications included variable oxidation of methionine, N-terminal acetylation, deamidation (N,Q) and fixed cysteine derivation by carbamidomethylation. The false discovery rate was set to <1%. Compilation of non-redundant protein lists was carried out using the Protein Extractor of the ProteinScape server (version 3, Bruker Daltonik GmbH, Bremen, Germany). Ion scores of individual MS/MS spectra lower than 30 and Mascot protein scores lower than 100 were not considered for the compilation of the identified proteins.

### 2.5 Bioinformatics analysis

The final list of identifications includes the proteins that are detected in at least three of the four OMV preparations, which were analyzed. This list contains 151 proteins, which are sorted according to their Clusters of Orthologous groups (COG) categories. The COG groups were created manually using the complete list of gene identifiers of the genome of strain SC1083 for batch searches in the COG database at National Center for Biotechnology Information (NCBI) at the URL: http://www.ncbi.nlm.nih.gov/Structure/cdd/wrpsb.cgi. The COG classifiers obtained by these searches were grouped according to the definitions provided in the NCBI conserved domains database [11]. The subcellular locations of the identified proteins were predicted using the program PSORTb 3.0 [12], and members of KEGG pathways were identified using the KOBAS 2.0 server [13] and the annotations of the *A. actinomycetemcomitans* strain D7S genome as a template.
