# Supplementary material for: Dataset of the proteome of purified outer membrane vesicles from the human pathogen Aggregatibacter actinomycetemcomintans
Source: Data Brief. 2016 Dec 15;10:426–31. doi: 10.1016/j.dib.2016.12.015 (PMC5192097; doi:10.1016/j.dib.2016.12.015)
Supplement: Supplementary file 1 — Supplementary material [file mmc1.docx]

**Conflict of interests**

The authors declare no conflict of interests.
